# Association of Parasitic Infections and Neurotropic Vitamins With Developmental Delay in Children With Severe Acute Malnutrition in Datia District, Madhya Pradesh: A Cross-Sectional Observational Study

**DOI:** 10.7759/cureus.115596

**Published:** 2026-09-01

**Authors:** Nidhi Sharma, Abhishek Sharma, Yisha Chandrakar, Pushpendra Singh, Suyesh Shrivastava, Anuradha Agarwal

**Affiliations:** 1 Microbiology, Government Medical College, Datia, Datia, IND; 2 Biochemistry, Government Medical College, Datia, Datia, IND; 3 Microbial Genomics, Indian Council of Medical Research (ICMR) - National Institute for Tribal Health Research (NITHR), Jabalpur, IND; 4 Epidemiology and Public Health, Indian Council of Medical Research (ICMR) - National Institute for Tribal Health Research (NITHR), Jabalpur, IND

**Keywords:** developmental delay, holotc, neurotropic vitamins, parasitic infestation, sam

## Abstract

Background and objective

Severe acute malnutrition (SAM) remains a significant health challenge among children under five in India and is often complicated by parasitic infestations and deficiencies of neurotropic vitamins. These factors may contribute to developmental delay. This study assessed the association between parasitic infestations and neurotropic vitamin deficiencies with developmental delay in children with SAM in Datia district, Madhya Pradesh, India.

Methods

This hospital-based, cross-sectional observational study was conducted at the Nutritional Rehabilitation Centre (NRC) of the Government Medical College and its associated hospital in Datia district, Madhya Pradesh, India, from April 2022 to April 2024. Stool microscopy and blood investigations were performed to identify parasitic infections and estimate vitamin B1, B6, and holotranscobalamin (HoloTC) levels. Developmental status was assessed using the Bayley Scales of Infant and Toddler Development, Fourth Edition (Bayley-IV). Data were analyzed using chi-square tests and logistic regression.

Results

Parasitic infections were detected in 30 (30%) children, with *Ascaris lumbricoides* identified in 10 (33%). Developmental delay was observed in 25 (71%) children with parasitic infections compared to 10 (29%) without infection. Mean vitamin levels were lower in infected than in non-infected children: vitamin B1 (0.132 ng/mL vs. 0.98 ng/mL), vitamin B6 (0.142 ng/mL vs. 0.768 ng/mL), and HoloTC (17 pmol/L vs. 27 pmol/L). On multivariate analysis, parasitic infection (adjusted odds ratio (aOR): 18.9, 95% CI: 5.2-68.2, p < 0.001) and HoloTC deficiency (aOR: 3.2, 95% CI: 1.05-9.7, p = 0.041) were independent predictors of developmental delay.

Conclusions

Parasitic infections and neurotropic vitamin deficiencies were strongly associated with developmental delay in children with SAM. Routine screening, deworming, and targeted nutritional interventions are essential to improve developmental outcomes in this vulnerable population.

## Introduction

Malnutrition is an alarming condition, leading to high rates of mortality and morbidity among children under five years of age in developing countries such as India [1]. The World Health Organization (WHO) defines severe acute malnutrition (SAM) as weight-for-height/length below −3 standard deviation (SD) of the WHO Child Growth Standards median, or a mid-upper arm circumference below 115 mm. Children with SAM are often immunodeficient, making them more vulnerable to parasitic infestations [1]. The prevalence of SAM in India is 7.7% according to the National Family Health Survey (NFHS-5). In Madhya Pradesh, the prevalence is 6.6% [2]. Among children under five years of age in India, the prevalence of parasitic infections is around 17% [3], while in the geographic area of Datia, the prevalence has been reported to be 21% [4].

Neurotropic vitamins such as B1, B6, and active B12 (holotranscobalamin (HoloTC)) are pivotal in the development of the central and peripheral nervous systems in children under five years of age. Children with SAM are deficient in these essential vitamins, and parasitic infestations further exacerbate these deficiencies, leading to developmental delays. Children living in unhygienic environments and exposed to adverse climatic conditions often suffer from acute malnutrition. When co-infected with parasites, their prognosis can worsen significantly if adequate interventions are not promptly instituted.

Overall growth and developmental delay, including fine motor delay, is reported to be three times more common in children with parasitic infections. In contrast, cognitive developmental delay is twice as common as in the general population [4].

Limited data are available from the Datia district regarding the association of parasitic infections and neurotropic vitamin deficiencies with developmental delay in children with SAM. Due to the limited information, physicians sometimes do not expect treatment responses in Nutritional Rehabilitation Centres (NRCs) in medical colleges and associated hospitals. Hence, this project was submitted to and approved by the Model Rural Health Research Unit (MRHRU), Datia. Preliminary data have been collected and are presented here to help guide necessary modifications in the management of children at NRCs. This study aims to address the challenges faced by malnourished children and provide evidence for targeted interventions.

The primary objective of this study was to determine whether parasitic infection and deficiencies of vitamin B1, vitamin B6, and HoloTC are associated with developmental delay among children with SAM admitted to the NRC in Datia. The secondary objectives were to describe the spectrum of parasitic infections and the distribution of neurotropic vitamin deficiencies in this population and to assess factors associated with developmental delay.

## Materials and methods

Study design and population

This hospital-based, cross-sectional observational study was conducted at the NRC of the Government Medical College and its associated hospital in Datia district, Madhya Pradesh, India, from April 2022 to April 2024.

Datia is one of the smallest districts of the state and has a predominantly rural population. According to the 2011 Census, the tribal population was 15,061 (1.91%), comprising 7,870 males and 7,191 females [5]. The study population consisted of children between 6 and 60 months of age admitted with SAM during the study period. According to WHO criteria [6], SAM was defined as weight-for-height/length below −3 standard deviations (SDs) of the WHO Child Growth Standards median or a mid-upper arm circumference (MUAC) less than 115 mm. The study protocol was approved by the Institutional Review Board (IRB) of Government Medical College, Datia, Madhya Pradesh, India (IRB Approval No.: 3218/IRB/GMC/Datia/2098801). Written informed consent was obtained from the caregivers of all participants.

Inclusion and exclusion criteria

Children aged 6-60 months who were diagnosed with SAM as per WHO guidelines and admitted to the NRC with caregiver consent were included in the study. Children with known congenital anomalies, major chronic systemic illnesses, or those on long-term medication likely to affect nutritional or neurodevelopmental status were excluded.

Sample size calculation

The sample size was calculated using a previously reported prevalence of intestinal parasitic infections of approximately 17% among malnourished children in India. A 95% confidence level and an absolute precision of 7.5% were used based on the study protocol and feasibility considerations. The calculated sample size was 96, which was rounded to 100 to account for potential non-response and ensure adequate coverage.

Data collection

Demographic and clinical details were recorded in a structured proforma during enrolment. Venous blood (3 mL) was collected in plain and ethylenediaminetetraacetic acid (EDTA) vials under aseptic precautions and processed in the central clinical laboratory. One milliliter of serum was separated and used for quantitative estimation of vitamin B1, vitamin B6, and HoloTC levels using enzyme-linked immunosorbent assay (ELISA) kits manufactured by Sunlong Biotech Pvt. Ltd. (Zhejiang, China). Optical densities were measured with an ELISA reader (Hydroflex TECAN, Austria). Peripheral blood smears were prepared and examined for malaria parasites. Stool samples were collected in sterile containers and examined by direct microscopy and the formalin-ethyl acetate concentration technique to detect intestinal protozoa and helminths. The concentrated specimens were examined microscopically for ova, cysts, and other parasitic forms. Parasites identified by microscopy were recorded based on their morphological characteristics. One stool specimen was collected from each participant for parasitological examination.

Developmental status was assessed using the Fourth Edition Bayley Scales of Infant and Toddler Development (Bayley-IV) [7]. This standardized tool evaluates cognitive, language, motor, social-emotional, and adaptive behavior domains. For this study, emphasis was placed on the motor domain to identify developmental delay associated with nutritional deficiencies and parasitic infections. Variables considered for analysis included age, sex, residence, type of parasitic infection, neurotropic vitamin levels (B1, B6, and HoloTC), and developmental delay. Formal permission to use the Bayley-IV was obtained from the copyright holder before conducting the study.

Statistical analysis

All data were entered into Microsoft Excel and analyzed using Statistical Package for the Social Sciences (SPSS) software (IBM Corp., Armonk, NY). Continuous variables were summarized as mean ± SD or median with interquartile range, depending on distribution, while categorical variables were presented as frequencies and percentages. Categorical variables were analyzed using the chi-square test or Fisher's exact test. A multivariable logistic regression analysis was performed to determine independent predictors of developmental delay. A p-value of <0.05 was considered statistically significant.

## Results

The study included 100 participants. Of these, 46 (46%) were aged 6-12 months, while 54 (54%) were aged 12-60 months. Regarding gender distribution, 44 (44%) participants were male, and 56 (56%) were female. With respect to residence, 55 (55%) participants belonged to rural areas, whereas 45 (45%) were from urban areas (Table 1).

**Table 1:** Sociodemographic distribution of patients (N = 100)

Variable	Total number, N (%)
Age	
6–12 months	46 (46%)
12–60 months	54 (54%)
Gender	
Male	44 (44%)
Female	56 (56%)
Residence	
Rural	55 (55%)
Urban	45 (45%)

Among the 30 children with SAM and parasitic infection, *Ascaris lumbricoides* was detected in 10 (33%), *Entamoeba spp*. in 6 (20%), *Giardia lamblia* in 5 (17%), hookworm in 3 (10%), *Plasmodium* species in 3 (10%), *Hymenolepis nana* in 1 (3%), *Trichuris trichiura* in 1 (3%), and *Strongyloides stercoralis* in 1 (3%) (Table 2).

**Table 2:** Pattern of parasitic infection in children with SAM SAM: Severe acute malnutrition.

Type of parasite	Total number, N (%)
Ascaris lumbricoides	10 (33%)
Giardia lamblia	5 (17%)
*Entamoeba *spp	6 (20%)
Hymenolepis nana	1 (3%)
Trichuris trichiura	1 (3%)
Strongyloides stercoralis	1 (3%)
Hookworm	3 (10%)
*Plasmodium *species	3 (10%)
Total	30 (100%)

Of the 30 infected children, monoparasitism was observed in 24 (80%), which included nematodes in 15 (50%), protozoa in 5 (17%), and cestodes in 4 (13%). Polyparasitism was present in 6 (20%), consisting of protozoa with nematodes in 3 (10%), nematodes with cestodes in 2 (7%), and protozoa with cestodes in 1 (3%). No significant association was observed (χ² = 2.21, p = 0.33) (Table 3).

**Table 3:** Distribution of SAM with the type of parasitism SAM: Severe acute malnutrition.

Type of infection	Classification of parasite	Number of cases, N (%)	Chi-square	p-value
Mono parasitism	Protozoa	5 (17%)	2.21	0.33
	Nematodes	15 (50%)		
	Cestodes	4 (13%)		
Poly parasitism	Protozoa and nematodes	3 (10%)		
	Nematodes and cestodes	2 (7%)		
	Protozoa and cestodes	1 (3%)		
Total		30 (100%)		

Developmental delay was more frequent in children with parasitic infection, occurring in 25 (71%) compared to 10 (29%) among those without disease. Mean vitamin levels were lower in infected children compared to non-infected children, with vitamin B1 at 0.132 ng/mL versus 0.98 ng/mL, vitamin B6 at 0.142 ng/mL versus 0.768 ng/mL, and HoloTC at 17 pmol/L versus 27 pmol/L (Table 4).

**Table 4:** Neurotropic vitamin levels and developmental delay among demographic subgroups Values of vitamin B1, vitamin B6, and holotranscobalamin (HoloTC) are expressed as mean ± standard deviation (SD) where available; in subgroups where dispersion measures were not calculated, values represent mean concentrations. The reference (normal) ranges for the studied parameters are as follows: vitamin B1, 16–48 ng/mL; vitamin B6, 4.1–48.7 ng/mL; and HoloTC, 40–200 pmol/L. The “Developmental delay” column represents the number (n) and percentage (%) of children identified with developmental delay in each subgroup, as assessed using Bayley’s Developmental Scale IV [7].

Variables	Vitamin B1, ng/mL (16-48)	Vitamin B6, ng/mL (4.1-48.7)	HoloTC, pmol/L (40-200)	Developmental delay
Age in months				
6-12 months	0.172 ± 0.014	0.297 ± 0.11	25.31 ± 4.89	10 (28.6%)
12-60 months	0.967 ± 0027	0.386 ± 0.23	29.23 ± 6.45	25 (71.4%)
Sex				
Male	1.45	0.967	23	17 (48.6%)
Female	1.03	0.872	21	18 (51.4%)
Residence				
Rural	1.56	0.83	23	16 (45.7%)
Urban	0.87	0.587	20	19 (54.3%)
Parasitic infection				
Positive	0.132	0.142	17	25 (71.4%)
Negative	0.98	0.768	27	10 (28.6%)

On multivariate logistic regression, parasitic infection emerged as a strong independent predictor of developmental delay, with an adjusted odds ratio (aOR) of 18.9 (95% CI: 5.2-68.2, p < 0.001). HoloTC deficiency was also significantly associated with developmental delay, with an aOR of 3.20 (95% CI: 1.05-9.70, p = 0.041). Other variables, including age 12-60 months (aOR: 2.25, 95% CI: 0.87-5.81, p = 0.093), vitamin B1 deficiency (aOR: 2.65, 95% CI: 0.89-7.92, p = 0.078), vitamin B6 deficiency (aOR: 1.85, 95% CI: 0.65-5.21, p = 0.25), male sex (aOR: 1.10, 95% CI: 0.44-2.75, p = 0.83), and urban residence (aOR: 1.45, 95% CI: 0.58-3.67, p = 0.42), were not statistically significant (Table 5).

**Table 5:** Multivariable logistic regression analysis of factors associated with developmental delay

Predictors	Adjusted odd ratios	95% CI	p-value
Age (12-60 months)	2.25	0.87–5.81	0.093
Male	1.10	0.44–2.75	0.83
Urban	1.45	0.58–3.67	0.42
Parasitic infection	18.9	5.2–68.2	<0.001
Vit B1 deficiency	2.65	0.89–7.92	0.078
Vit B6 deficiency	1.85	0.65–5.21	0.25
HoloTC deficiency	3.20	1.05–9.70	0.041

## Discussion

This study highlights the multifactorial role of nutritional deficiencies and parasitic infections in developmental delay among children with SAM. Several studies [8-13] have reported nutritional deficiencies, particularly neurotropic vitamin deficiencies, as well as parasitic infections in children with SAM. Our study examined the association between parasitic infections and neurotropic vitamin status and their combined effect on developmental milestones.

In children with SAM, parasitic infestations interfere with nutrient absorption and increase metabolic demand through chronic inflammation, further depleting nutritional reserves. Blood loss and reduced food intake worsen the condition. Malnutrition, in turn, compromises immune function, making children more susceptible to parasitic infections, thereby creating a vicious cycle.

Vitamin B12 is absorbed in the small intestine, while most gut microbiota are present in the colon. Although the small intestine is not sterile, its resident microbiota influence vitamin absorption [14-16]. Certain bacteria synthesize vitamin B12; however, children with parasitic infections, especially hookworm and *Giardia*, may have impaired production or absorption of this vitamin. Parasitic infections may adversely affect nutritional status through several mechanisms, including reduced nutrient intake, impaired intestinal absorption, blood loss, and chronic inflammatory responses. In the present study, children with parasitic infections had lower concentrations of vitamin B1, vitamin B6, and HoloTC than those without infection. These findings suggest an association between parasitic infection and micronutrient status in children with SAM; however, the cross-sectional design does not allow determination of the underlying mechanisms or causality.

In the present study, developmental delay and lower vitamin concentrations were more frequently observed among children aged 12-60 months, females, and those residing in rural areas. However, these subgroup differences were not statistically significant in the multivariable analysis. Therefore, these findings should be interpreted as descriptive patterns rather than independent associations. Our results are consistent with findings from studies in sub-Saharan Africa [2]. Females and younger children recently weaned from breast milk may have underdeveloped immune systems, poorer hygiene, and gender-related disparities in nutritional access, contributing to higher vulnerability. The prevalence of parasitic infections in our study was also higher than reported in Ethiopia and Sudan [2].

The high prevalence observed in this region underscores the importance of deworming in the management of children with SAM under five years of age. Improvements in water safety, food hygiene, and sanitation are also essential to reduce the burden of parasitic infections and malnutrition. The elevated prevalence may be related to differences in awareness, health education, and local environmental conditions.

In our study, the concentrations of vitamin B1, B6, and HoloTC in children aged 6-12 months were 0.172 ± 0.014 ng/mL, 0.297 ± 0.11 ng/mL, and 25.31 ± 4.89 pmol/L, respectively. Among children aged 12-60 months, the corresponding values were 0.967 ± 0.027 ng/mL, 0.386 ± 0.23 ng/mL, and 29.23 ± 6.45 pmol/L. Developmental delay was observed in 10 children younger than 12 months and 25 children aged 12-60 months. Our findings are consistent with those of Shalini et al. [8], who reported thiamine deficiency in 40% of children with SAM. Vitamin B6 deficiency was also more frequent in children aged 12-60 months, although data on this vitamin in parasitic infestations remain limited.

Neurotropic vitamin deficiency was more common in females than in males, consistent with the findings of El Elella​​​​​ et al. [9], but contrasting with Sharma et al. [10], who reported a higher prevalence in males. In our study, deficiencies were more common in the rural population, aligning with Vaid et al. [16], but contrasting with Shalini et al. [8]. Children with SAM and parasitic infections had significantly lower vitamin concentrations compared with those without infections, similar to the findings of Olivares et al. [12].

The overall incidence of parasitic infections among children with SAM was 30%. Protozoal infections accounted for 17%, mainly *Entamoeba *spp. (20%) and *Giardia lamblia *(17%). This prevalence was slightly higher than that reported by Gadisa et al. [13], possibly due to differences in hygiene awareness and geographical factors. Protozoal infection rates typically range from 2% to 7% in developed countries and 20% to 30% in developing countries, consistent with studies from Nigeria and Tanzania [14].

Nematodes constituted the majority of parasitic infections, observed in 50% of monoparasitic cases, and were also present in polyparasitic infections with protozoa (10%) and cestodes (7%). These findings are consistent with those of Gadisa et al. [13] and Ekeng et al. [14], although Mossman et al. [15] reported a higher proportion of non-parasitic cases.

The overall parasitic load was 30%, with 70% being monoparasitic infections. The most frequent helminth was *A. lumbricoides* (33%), followed by hookworm (10%), *T. trichiura* (3%), *H. nana* (3%), and *S. stercoralis* (3%). *Entamoeba​​​​ spp.* (20%) and *G. lamblia* (17%) predominated among protozoa, while *Plasmodium* species accounted for 10%. The high burden of *Ascaris* infections is consistent with findings from Mossman et al. [15]. The lower prevalence of *S. stercoralis* may be attributed to geographic or HIV-related differences. The prevalence of *Plasmodium* infection (10%) was nearly equal to that of hookworm infections. This is consistent with Ekeng et al. [14] but differs from reports by Mossman et al. [15] and Gadisa et al. [13]. The increased burden of malaria may be explained by poor environmental conditions, lack of vector control, and favorable breeding sites for *Anopheles *mosquitoes [16-22]. Environmental and hygienic factors likely contribute to fluctuations in parasitic load [23,24]. The predominance of *Entamoeba* *histolytica* (21%) in our study is similar to that reported by Gadisa et al. [13], whereas *G. lamblia* was more common in studies by Mossman et al. [15].

Limitations

This study has several limitations. First, its cross-sectional design precludes establishing a temporal or causal relationship between parasitic infection, micronutrient status, and developmental delay. Second, the study was conducted at a single hospital-based NRC, which may limit the generalizability of the findings to children with SAM in other settings. Third, the relatively small sample size may have limited the statistical power to detect some associations. In addition, although multivariable analysis was performed, residual confounding by factors such as dietary intake, socioeconomic status, and severity of malnutrition cannot be completely excluded. A larger multicenter comparative study would be useful for validating these findings and more firmly establishing the independent contributions of parasitic infection and micronutrient deficiencies to developmental delay.

## Conclusions

Parasitic infections and neurotropic vitamin deficiencies were common among children with SAM admitted to the NRC in Datia. Parasitic infection and reduced HoloTC levels were independently associated with developmental delay in the multivariable analysis. These findings suggest that screening for parasitic infections and micronutrient deficiencies may be clinically relevant when assessing children with SAM and developmental concerns. However, given the cross-sectional nature of the study, these associations should not be interpreted as causal. Larger, multicenter prospective studies are warranted to further clarify the temporal and causal relationships among parasitic infections, micronutrient status, and developmental outcomes in children with SAM.

## References

[1] World Health Organization (2025). Management of severe acute malnutrition in children 6-59 months of age with oedema. http://www.who.int/tools/elena/interventions/oedema-sam.

[2] Ulahannan SK, Wilson A, Chhetri D, Soman B, Prashanth NS (2022). Alarming level of severe acute malnutrition in Indian districts. BMJ Glob Health.

[3] Banerjee S, Ray S, Shrivastava P, Das DK (2020). Prevalence of intestinal parasitosis among under-five children in a rural community of Purba Bardhaman district, West Bengal. Indian J Community Med.

[4] Yogyata M (2011). Prevalence of intestinal parasites at Ujjain, Madhya Pradesh, India: five-year study. Afr J Microbiol Res.

[5] Office of the Registrar General & Census Commissioner (2025). District Census Handbook - Datia, Madhya Pradesh. https://censusindia.gov.in.

[6] World Health Organization (2025). WHO child growth standards and the identification of severe acute malnutrition in infants and children. https://www.who.int/publications/i/item/9789241598163.

[7] Bayley N, Aylward GP (2019). Bayley Scales of Infant and Toddler Development, Fourth Edition (Bayley-IV). https://www.pearsonassessments.com/en-us/Store/Professional-Assessments/Cognition-%26-Neuro/Bayley-Scales-of-Infant-and-Toddler-Development-%7C-Fourth-Edition/p/100001996?srsltid=AfmBOorzZ9JyzoRC4m8Ocvb6gVsXqdHl1kvYYs6f98EMfhl16k1jeEDt.

[8] Shalini T, Pullakhandam R, Ghosh S (2023). Prevalence of vitamin B12 and folate deficiencies in Indian children and adolescents. Nutrients.

[9] El Elella SSA, Tawfik MA, El Fotoh WMA, Barseem NF (2017). Screening for developmental delay in preschool-aged children using parent-completed ages and stages questionnaires: additional insights into child development. Postgrad Med J.

[10] Sharma AR, Siddiqui MS, Magar S, Kale A, Nelanuthala M, Singh SP (2023). The etiological profile of global developmental delay at a tertiary care hospital in India: an observational study. Cureus.

[11] Ganesh K, Stadler ZK, Cercek A, Mendelsohn RB, Shia J, Segal NH, Diaz LA (2019). Immunotherapy in colorectal cancer: rationale, challenges and potential. Nat Rev Gastroenterol Hepatol.

[12] Olivares JL, Fernández R, Fleta J, Ruiz MY, Clavel A (2002). Vitamin B12 and folic acid in children with intestinal parasitic infection. J Am Coll Nutr.

[13] Gadisa E, Jote K (2019). Prevalence and factors associated with intestinal parasitic infection among under-five children in and around Haro Dumal Town, Bale Zone, Ethiopia. BMC Pediatr.

[14] Ekeng B, Adedokun O, Otu V (2024). The spectrum of pathogens associated with infections in African children with severe acute malnutrition: a scoping review. Trop Med Infect Dis.

[15] Mosmann TR, Coffman RL (1989). Heterogeneity of cytokine secretion patterns and functions of helper T cells. Adv Immunol.

[16] Vaid A, Sharma M, Jamunashree B, Gautam P (2018). Serum vitamin B12 levels in severe acute malnutrition hospitalized children between age group 6 months to 59 months in Kangra, India. Int J Contemp Pediatr.

[17] Warghat PA, Sharath HV, Raghuveer R (2024). The effect of early pediatric rehabilitation in an infant with vitamin B12 deficiency associated with developmental delay: a case report. Cureus.

[18] Harel Y, Zuk L, Guindy M, Nakar O, Lotan D, Fattal-Valevski A (2017). The effect of subclinical infantile thiamine deficiency on motor function in preschool children. Matern Child Nutr.

[19] Adhualia A, Maurya M, Tewari DA (2019). Developmental delay in children with severe acute malnutrition and its association with vitamin B12 deficiency. Int J Contemp Pediatr.

[20] Shrestha BK, Tumbahangphe M, Shakya J (2021). Prevalence and related risk factors of intestinal parasitosis among private school-going pupils of Dharan submetropolitan city, Nepal. J Parasitol Res.

[21] Mustafa UK, Zacharia A, Mushi V (2021). Prevalence of intestinal protozoa infections and associated risk factors among preschool aged children in Mkuranga District, Tanzania: a community based cross-sectional study. Tanzan J Health Res.

[22] von Huth S, Kofoed PE, Holmskov U (2019). Prevalence and potential risk factors for gastrointestinal parasitic infections in children in urban Bissau, Guinea-Bissau. Trans R Soc Trop Med Hyg.

[23] Mengist HM, Zewdie O, Belew A (2017). Intestinal helminthic infection and anemia among pregnant women attending ante-natal care (ANC) in East Wollega, Oromia, Ethiopia. BMC Res Notes.

[24] Yesuf DA, Abdissa LT, Gerbi EA, Tola EK (2019). Prevalence of intestinal parasitic infection and associated factors among pregnant women attending antenatal care at public health facilities in Lalo Kile district, Oromia, Western Ethiopia. BMC Res Notes.

